# Variation in salinity tolerance between and within anadromous subpopulations of pike (*Esox lucius*)

**DOI:** 10.1038/s41598-017-18413-8

**Published:** 2018-01-08

**Authors:** Johanna Sunde, Carl Tamario, Petter Tibblin, Per Larsson, Anders Forsman

**Affiliations:** 1 Ecology and Evolution in Microbial Model Systems, EEMiS, Department of Biology and Environmental Science, Linnaeus University, SE-391 82 Kalmar, Sweden; 2 Present Address: Swedish University of Agricultural Sciences, Department of Aquatic Resources, SE-702 15 Örebro, Sweden

## Abstract

Environmental heterogeneity is a key determinant of genetic and phenotypic diversity. Stable and homogenous environments tends to result in evolution of specialism and local adaptations, while temporally unpredictable environments may maintain a diversity of specialists, promote generalist strategies, or favour diversified bet hedging strategies. We compared salinity tolerance between two anadromous subpopulations of pike (*Esox Lucius*) that utilize freshwater spawning sites with different salinity regimes. Eggs from each population were artificially fertilized and incubated in a salinity gradient (0, 3, 5, 7, and 9 psu) using a split-brood design. Effects on embryonic development, hatching success, survival of larvae, and fry body length were compared between populations and families. The population naturally spawning in the stable freshwater habitat showed signs of specialization for freshwater spawning. The population exposed to fluctuating selective pressure in a spawning area with occasional brackish water intrusions tolerated higher salinities and displayed considerable variation in reaction norms. Genetic differences and plasticity of salinity tolerance may enable populations to cope with changes in salinity regimes associated with future climate change. That geographically adjacent subpopulations can constitute separate units with different genetic characteristics must be considered in management and conservation efforts to avoid potentially negative effects of genetic admixture on population fitness and persistence.

## Introduction

How environmental heterogeneity shapes biological diversity and the different ways by which individuals, populations and species cope with changing conditions are overarching questions in evolutionary ecology. In environments that are relatively stable and homogeneous, natural selection tends to favour those genotypes and phenotypes that are beneficial under the most prevalent conditions, resulting in evolution of specialist strategies and local adaptations^1,2^. Under spatial heterogeneity, divergent selection may result in evolution of different local adaptations in different areas and contribute to the maintenance of greater genetic variation in the species as a whole^3–5^. In environments that change over time, selection may favour different genotypes and phenotypes during different periods^6,7^. This may maintain a diversity of specialists within populations, or promote the evolution of generalist strategies that perform reasonably well across a range of environments^1,7,8^. Generalist strategies can also consist of plastic or flexible phenotypes that adjust to conditions via developmental modifications (e.g., phenotypic plasticity) or via reversible intra-individual behavioural (e.g., matching habitat choice) or physiological (e.g., colour change, osmoregulation) modifications^9–12^. Fluctuating unpredictable environments may also favour a diversified bet hedging strategy and individuals that produce an optimal mix of different specialists within a single batch of progeny^13,14^. Insights into these complex issues may ultimately improve projections regarding how populations, species, and ecosystems will respond to shifting selection pressures brought about by altered land use and climate change^15–17^.

Models project that future climate change will affect hydrogeography, water temperatures, and salinity in aquatic systems^15,18^. Salinity fluctuations in time and space play an important role in defining the distribution of aquatic organisms, including many fish species^19,20^. Teleost fishes use osmoregulation to keep internal ion concentration constant. Fishes may live their entire lives within one salinity regime. Diadromous fishes however, such as Atlantic and Pacific salmon^21^, freshwater *Anguilla spp*. eels^22^, the *Gasterosteus aculeatus* stickleback species complex^23^, and anadromous populations of pike (*Esox Lucius*) in the Baltic Sea^24,25^, migrate and utilize different salinity regimes during certain parts of life.

Pike, *Esox lucius*, is an iteroparous, large (<130 cm) and long-lived (>10 years) fish species with a circumpolar distribution on the northern hemisphere where it occupies lakes, rivers and brackish waters^26–28^. It is also a valuable model organism for studies in ecology and evolutionary biology^29^. Pike is of freshwater origin but has been found to tolerate moderate levels of brackish water, most notably in the Baltic Sea which is one of the largest brackish water (estuarine) areas on earth^25,27,30^. It inhabits the majority of the Baltic Sea coastal areas, from the Bothnian Bay in the north, to the Baltic Proper in the west, where the saline water originating from the North Sea^31^ limits its distribution. While adult pike can cope with saline water up to 12–15 ppt^30^ Jacobsen (2007)^32^ report that pike fry do not survive salinities over 13 ppt, and Jørgensen *et al*.^33^ reported symptoms of severe stress for brackish water pike fry at salinities above 13 psu.

**Figure 1:**
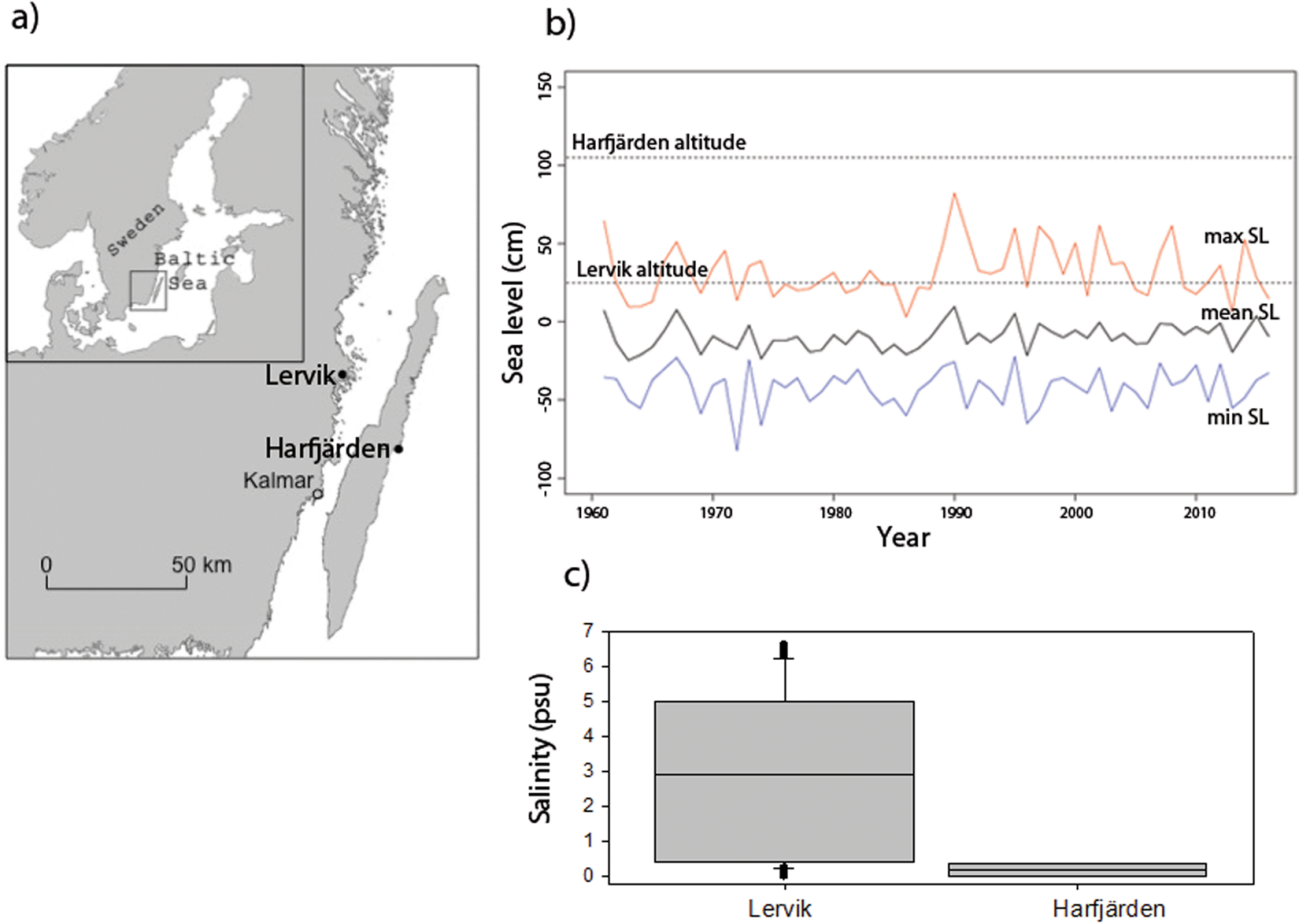
Locations and altitides of study locations, Baltic Sea level fluctuations and salinity regimes. (**a**) Map of spawning sites for anadromous *Esox lucius* included in the experiment. The map was generated in ArcMap version 10. http://desktop.arcgis.com/en/arcmap/. The figure was prepared in Adobe Photoshop CS5 Extended, version 12.0.4 × 32, http://www.adobe.com/se/products/photoshop.html. (**b**) Altitude in centimeters above reference sea level for the two wetlands, and sea levels outside each wetland during pike spawning time (March to June) during the period 1961–2015. The dashed black lines indicate the mean altitudes of the two wetlands, the black solid line the mean sea level during spawning time, the blue line the lowest measured sea level during spawning time, and the red line the highest measured sea level during spawning time. **(c)** Salinity (psu) of water samples collected manually in the Lervik and Harfjärden wetland spawning areas between March 1 – May 31, during 1994 to 2017.

Pike in the Baltic Sea employ two reproductive strategies that differ with regard to salinity regimes. Spawning can occur directly in brackish waters along the coast^25,34^. However, about 50% of the pike found off the south eastern coast of Sweden in the Baltic Sea are anadromous and migrate from brackish to freshwater to spawn^24,28^. In our study area (Fig. 1), most anadromous pike juveniles stay less than one month in freshwater before emigrating to the Baltic Sea^35^, where they spend the main part of the year living sympatrically with other subpopulations of anadromous and brackish spawning pikes. Previous studies suggest that freshwater spawning pike are unable to produce offspring in brackish waters over 7 ppt^25,30^. Conversely, a brackish spawning population seems to have lost its ability to spawn in freshwater^33^. While this suggests that adaptation to one salinity environment may come at the cost of impaired performance in other environments, little is known about whether there exists genetically encoded variation or plastic responses that can buffer against environmental change.

The strong natal homing behaviour^12,36^ has allowed for anadromous pike subpopulations that spawn in different but adjacent streams and wetlands to become genetically separated^28^. It has also allowed evolution of local adaptations of morphological and life history traits (*e*.*g*. growth rate, body size, and vertebral count)^37–39^. However, it is not known whether the anadromous subpopulations of pike differ in salinity tolerance, or whether they harbour sufficient standing genetic variation or have capacity for phenotypic plasticity enabling them to cope with changes in salinity associated with seasonality or longer term climate change.

The objective of this study was to compare salinity tolerance of two genetically distinct anadromous subpopulations of pike from the Baltic Sea that utilize freshwater spawning environments with contrasting salinity regimes (Fig. 1). To test for differences in salinity tolerance, a split-brood experiment in a salinity gradient (0, 3, 5, 7 and 9 psu) was conducted, and effects on embryonic development, hatching success, growth and survival of larvae were compared between populations and among families. Furthermore, to test for genetic variation in plasticity, the gene by environment reaction norm (GxE) linking performance to salinity was compared among families within populations as well as between populations.

We hypothesized that the Harfjärden population spawning in the stable freshwater wetland should have a lower tolerance to salinity overall and exhibit less genetic variation for salinity tolerance, that is a higher degree of specialisation, compared with the Lervik population. The Lervik population spawning in the wetland occasionally influenced by backflow of brackish water was instead predicted to show signs of generalism that manifest as greater diversity in salinity tolerance either among families (different specialists) or within families (generalists).

## Methods

### Study populations

Lerviksbäcken (henceforth Lervik, N 57° 04.414′; E 16° 31.246′) is located on the Swedish mainland east coast, and Harfjärden (N 56° 49.063′; E 16° 48.673′) is located on the east coast of the island of Öland (Fig. 1). In a previous study Larsson and colleagues^28^ analysed genetic variation based on 10 nuclear microsatellite DNA loci (Elu 2, Elu, 6, Elu 19, Elu 37, Elu 51, Elu 64, Elu 76, Elu 78, Elu 86, Elu 276 (Miller and Kapuscinsky 1996^40^; 1997^41^; Hansen *et al*. 1999^42^)) and found a strong neutral genetic differentiation between the populations spawning in Lervik and Harfjärden (pairwise *F*_*ST*_ = 0.226), despite geographic proximity^28^. This suggests that gene flow is probably too weak to prevent evolution of local adaptations. Within population neutral genetic diversity was slightly lower for Lervik (gene diversity 0.324 ± 0.187 *N* = 36) than for Harfjärden (0.456 ± 0.251, *N* = 41)^28^.

The spawning locations differ in their salinity regimes. The stream leading up to the Lervik wetland flows through agricultural land at a very low inclination, and downstream water flux is limited to floods in the spring and rainy periods in addition to being heavily dependent on the water level in the Baltic Sea^43^. The spawning area in Lervik is in level with the altitude of the Baltic Sea, and the water level in the sea periodically exceeds that of Lervik (Fig. 1b), allowing backflow of brackish water to enter and temporarily increase salinity in the spawning area (occassionaly up to 7 psu, see Fig. 1c). By contrast, the spawning area in Harfjärden on Öland is fed solely by freshwater and located at an altitude 1 m above the Baltic Sea water level (Fig. 1b), such that it cannot be influenced by backflow of brackish water. In regards to salinity, the two spawning locations thus represent contrasting levels of environmental heterogeneity (Fig. 1c).

Altitude analysis was performed in ArcMap version 10 using geodata (2-meter resolution raster) from the Swedish Authority for Geographic and Geometric Information (Lantmäteriet). Twenty-five height measurement points were placed at random within each wetland and outside the outlet of each wetland (in total 100 datapoints). The wetland in Harfjärden was located 1.07 meters (±0.02 SD) above mean sea level (MSL) and the wetland in Lervik was at 0.24 meters (±0.02 SD) above MSL. Sea level analysis was performed in RStudio version 0.99.903 using hourly measured sea level data for Oskarshamn (years 1961 to 2016) from the Swedish Meteorological and Hydrological Institute (SMHI).

### Collection of parental fish, eggs and milt

Adult mature pikes were caught at the inlets of each spawning location using fyke nets. Eleven females and eleven males from each of the two populations (in total *N* = 44 parents) were haphazardly selected, and stripped for gametes on April 5 and April 6, 2016. Both locations were sampled simultaneously in both days, and populations contributed an equal number of parental individuals and amount of gametic material each day. There was no difference in body length between females collected from Harfjärden (mean ± S.D., 67 ± 5.5 cm) or Lervik (69 ± 10.4 cm, *t* = 0.72, *df* = 20, *P* = 0.48). The eggs and milt were collected in individual tubes (50 ml falcon tubes for eggs and 2 ml Eppendorf tubes for milt) and placed on ice until artificial fertilization commenced in a laboratory environment (see below). The stripped individuals were returned to their natural spawning habitat immediately after handling.

### Salinity tolerance experiment

A split-brood salinity tolerance experiment was conducted at Kalmarsund Laboratory of Linnaeus University in Kalmar, Sweden. The experiment was carried out in a constant room, set such that water temperature remained stable at around 12 °C, with a (15 L: 9D) light regime. This setup was chosen to resemble natural conditions, and to ensure that the temperature was within the natural range at spawning^35,44^. Within each population, families were created by randomly assigning each male to a female. Each population and family was tested in five salinities (0, 3, 5, 7 and 9 psu), with two replicates per family and salinity treatment, resulting in a total of 220 experimental units (2 populations, 11 families (female/male pairs) per population, 5 salinity treatments per family, 2 replicates per family and treatment). The salinity gradient range was chosen to include and slightly exceed the levels in the wetlands used for spawning (Fig. 1c) and that of the Baltic Sea. All fertilizations occurred between one female and one male from the same population to enable detection of any family effects and to allow tests for family (genotype) by environment (salinity) interaction effects (GxE). The water used for the experiment was tap water that had been aerated for at least 3 hours. The five levels of salinity were acquired through mixing batches of 10 litres of aerated tap water with Sera Marin Sea Salt.

Experimental units were prepared as follows. Thirty eggs from a single female were placed in a small glass cup and inundated in an excess of milt obtained from one male originating from the same population as the female. Fertilization was induced by adding water of 0, 3, 5, 7 or 9 psu, depending on salinity treatment, in a volume approximately equal to the eggs. The female and male gametes were gently mixed, and then eggs were allowed to rest for two minutes before rinsing them with water of the corresponding salinity to rid the eggs of excess milt^38,39^. The fertilized eggs were then carefully transferred to a plastic incubation chamber (hereafter bin) with the corresponding salinity treatment. A bin was assembled from two food approved, circular 800 mL plastic cups. One cup was inserted into the other, and the bottom of the inner cup had been replaced with a net (made of plastic with a mesh size of 1.5 × 1.5 mm) on which the eggs were allowed to rest.

The 220 experimental units were placed randomly on shelves in the laboratory to minimize any systematic effects associated with differential airflow, temperature or light in the room. Aerated tap water was added to the bins on two occasions during the experimental period to compensate for evaporation. Additionally, during the experimental period, partial water exchanges with treatment specific salinities were carried out two times, and a complete water exchange one time. Prior to each of the three water exchanges, aerated tap water was added to the bins to compensate for evaporation, thus compensation for evaporation was added five times in total. Eggs were inspected and dead eggs were removed from aquaria with a plastic pipette. The experiment was terminated after 21 days. Photographs (using Panasonic DMC-TZ5) of each experimental unit showing eggs and larvae were taken daily, commencing three days after fertilization and continuing until termination. Live brine shrimp (*Artemia salina*) were provided as food *ad libitum* three times per day starting 7 days post hatch and until termination of the experiment.

### Collection of data

**Figure 2:**
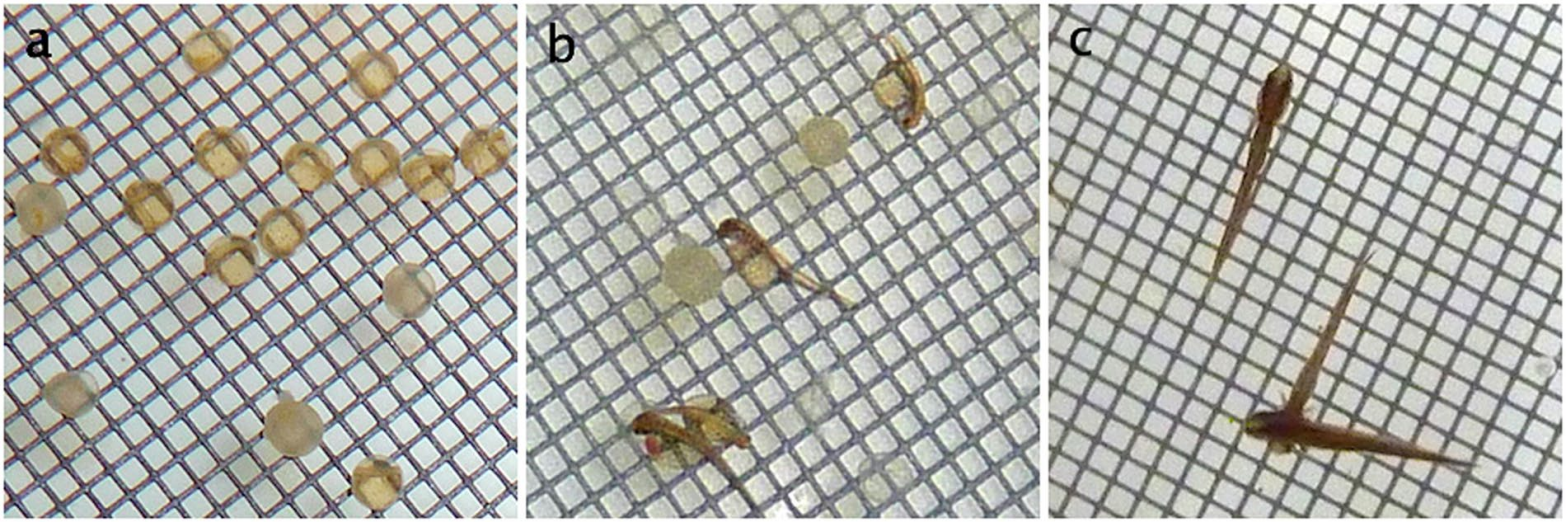
Development of *Esox lucius* eggs. (**a**) Eggs with developing embryos at day 7. (**b**) Newly hatched yolk-sac larvae at day 10. (**c**) Surviving fry at day 21. Photos by Carl Tamario.

Data sampling was done through analyzing the series of digital photographs that was obtained for each experimental unit and included counting of (i) total number of eggs, (ii) eggs with successful embryonic development, (iii) time to hatching, (iv) successfully hatched larvae, (v) survival of larvae through the yolk sac phase, as well as digital measurement of (vi) fry body length at the end of the yolk sac phase (Fig. 2).

The first assessment of egg survival, signs of successful embryonic development, was performed seven days after experimental onset and the mixing of gametes, when a discernible embryo had started to develop (Fig. 2a). At this time, the embryos have darkened slightly and can be easily discerned by ocular inspection. All embryos that had developed past the spherical shape and started to stretch out into larval shape were classified as hatched (Fig. 2b). Time of hatching for each unit was specified as the day when > 50% of the eggs in the bin had hatched. Hatching success was quantified as the total number of hatched larvae divided by the total number of eggs. Survival through the hatching process (between day 7 and > 50% hatched) was quantified as the total number of hatched larvae divided by the number of visible embryos. Survival of larvae through the yolk sac phase was quantified by dividing the number of live fry at termination by the total number of hatched larvae.

At termination of the experiment, all fry were photographed against the cage bottom mesh (Fig. 2c). Measurements of body length were obtained from the digital images using the free image editing software ImageJ.

Eggs that fail to be fertilized during the activation window (according to Raat^30^ micropyle stays open for approximately 1 minute) die, and dead eggs become milky and are easily discernible (Figure S1a). However, results from a complementary incubation experiment showed that the timing of the morphological appearance of dead eggs differed between salinities (Supporting methods and results, Figure S1b), such that accurate assessments and comparisons of fertilization success through inspection of eggs at an early stage was not possible.

### Statistical analysis

To test if embryonic development, hatching success of eggs, survival of larvae, and fry body length varied among source populations, was affected by salinity, and whether the effect of salinity depended on source population we performed general linear mixed models analysis of variance, GLMM, implemented using the procedure MIXED in SAS^44,45^. Population and salinity were treated as fixed factors, and the interaction between population and salinity was included to evaluate whether the relationship between the response variable and salinity was different in the two populations. Family (female/male pair) was included as a random factor to model and take into account any maternal effects and the covariance of repeated measures on the same family (two replicates for each female and salinity level). However, we were not interested in evaluating or quantifying effects of family as such. Parameter estimates and statistical significance levels associated with family are therefore not reported in the tests for effects of population and salinity (except when assessing family by environment (GxE) interactions, see below).

A major benefit of the MIXED procedure is that it can handle unbalanced data^44^, such as when repeated measures are not available for all families, e.g., due to losses from mortality (as in the present study). We performed separate analyses for each dependent (response) variable. Data on proportion of eggs that initiated embryonic development, proportion embryonic eggs that hatched, and proportion hatched larvae that survived through the yolk sac phase were arcsine-square root transformed to meet the assumptions of normality and and homogeneity of variances. Models were fitted to the data using restricted maximum likelihood method, and the Kenward-Roger method was used to approximate degrees of freedom^45^.

Results and conclusions regarding effects of source population and salinity on embryonic development, hatching success and survival of fry remained unchanged, with one exception (see below), when data were analysed in their original binary form (instead of using transformations of proportions) by applying generalized linear mixed models (GLMMs) with a binomial fit and a logit-link power function, using procedure GLIMMIX in SAS. The single case in which the two statistical approaches generated qualitatively different results concerned the analyses of hatching success of eggs that had developed into the embryonal stage. The results based on analysis of binary data using GLIMMIX indicated a statistically significant effect of the interaction between population and salinity treatment (*F*_4,135_ = 3.63, *P* = 0.0076). The results based on analyses of proportions instead indicated that the effect of the interaction was not statistically significant, and we adhere to this more conservative outocome and interpreation in the results below.

To assess within population diversity and test for effects of gene by environment interactions (crossing norms of reaction, GxE) on hatching success, data for each population was analysed separately using procedure MIXED, and the interaction between family and salinity was treated as a random factor. Statistical significance of the random factor was assessed using the Wald *Z* test^44^.

To evaluate whether the variance in salinity tolerance of hatching success and body length of fry at the end of the yolk sac phase differed between the two populations we first computed the total variance among and within (two replicates per family and salinity) families for each population and salinity level. Next we used ANCOVA to test for an effect of source population on total variance while statistically controlling for any potential effects of salinity. Hypotheses were tested against an α–level of 0.05.

### Data availability

The datasets generated during and/or analysed during the current study are available from the corresponding author on reasonable request.

## Results

**Figure 3:**
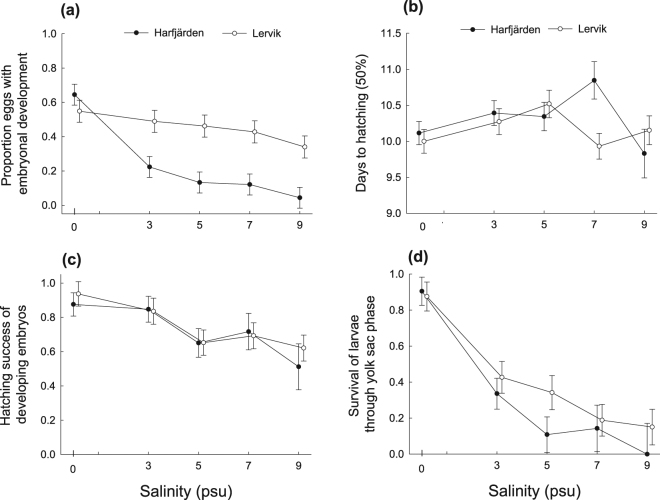
Effects of salinity on performance of eggs and larvae in two subpopulations of *Esox lucius*. **(a)** The proportion of eggs that produced live embryos after seven days. (**b**) Days until at least 50% of the eggs had hatched. (**c**) Hatching success of eggs with developing embryos after seven days. (**d**) Survival of hatched larvae through the yolk sac phase. Figure shows least-squares means ± s.e. Data points for the Harfjärden population have been offset along the horizontal axes to visualize overlapping observations.

The proportion of eggs that developed to the embryonal phase decreased with increasing salinity, but the response to salinity was different in the two populations (GLMM, effect of population: *F*_1,17_ = 10.08, *P* = 0.0055; effect of salinity: *F*_4,163_ = 32.21, *P* < 0.0001; effect of two-way interaction between population and salinity: *F*_4,163_ = 10.52, *P* < 0.0001). The two populations responded similarly to freshwater but the incidence of developing embryos declined with increasing salinity at a faster rate in the Harfjärden population (Fig. 3a).

Average time until 50% of the eggs in each bin had hatched was 10.22 days (±0.69 SD, *n* = 131) and did not vary significantly according to population, salinity or their interaction (GLMM, effect of population: *F*_1,21.1_ = 0.69, *P* = 0.4140; effect of salinity: *F*_4,113_ = 2.13, *P* = 0.0818; effect of two-way interaction between population and salinity: *F*_4,113_ = 2.43, *P* = 0.0515) (Fig. 3b).

Hatching success of eggs that had developed into the embryonal stage was high (ca 90%) in freshwater, decreased with increasing salinity and did not differ significantly between source populations (GLMM, effect of population: *F*_1,17.5_ = 0.04, *P* = 0.84; effect of salinity: *F*_4,125_ = 5.06, *P* = 0.0008; effect of two-way interaction between population and salinity: *F*_4,125_ = 0.26, *P* = 0.9007) (Fig. 3c).

Survival of hatched larvae through the yolk sac phase was high (ca 90%) in freshwater, decreased with increasing salinity and did not differ significantly between source populations (GLMM, effect of population: *F*_1,121_ = 2.54, *P* = 0.1135; effect of salinity: *F*_4,121_ = 23.44, *P* < 0.0001; effect of two-way interaction between population and salinity: *F*_4,121_ = 0.45, *P* = 0.7710) (Fig. 3d).

**Figure 4:**
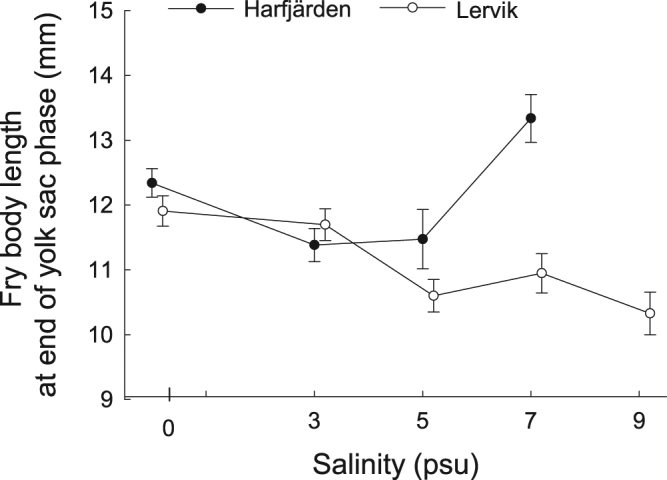
Body length as a function of salinity. Relationship between mean body length (in millimetres) of live fry at the end of the yolk sac phase and rearing salinity in *Esox lucius* populations from Harfjärden and Lervik. Figure shows least-squares means ± s.e.

Body length of fry at the end of the yolk sac phase was influenced by salinity and the response to salinity differed between populations (GLMM, effect of population: *F*_1,22.1_ = 6.10, *P* = 0.0217; effect of salinity: *F*_4,840_ = 19.56, *P* < 0.0001; effect of two-way interaction between population and salinity: *F*_3,851_ = 15.59, *P* < 0.0001). Overall, fry from Lervik decreased in length with increasing salinity (regression based on least-squares means shown in Fig. 4, *F*_1,3_ = 13.02, *P* = 0.0366) whereas no such trend was recorded in the Harfjärden population (*F*_1,2_ = 0.28, *P* = 0.648) (Fig. 4).

### Family-by-environment (GxE) interaction effects on hatching success and fry length

**Figure 5:**
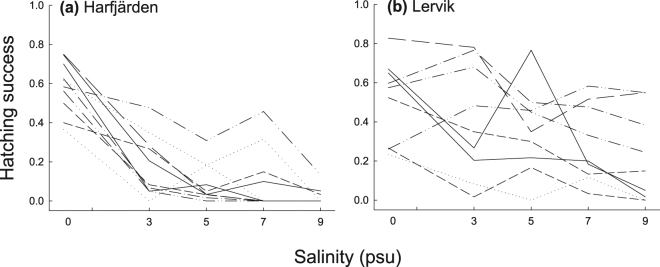
Genotype by environment interactions (GxE) linking hatching success to salinity. Variation within and among families in how hatching success of eggs responded to salinity in *Esox lucius* populations from (**a**) Harfjärden and (**b**) Lervik. Each line represents data for one split-brood family of full siblings reared under five different salinities. Figure is based on means of two replicates within each family and salinity treatment.

The responses to salinity varied among families within populations, thus indicating genetic variation in reactions norms (GxE) within both study populations for hatching success of eggs (Table 1, Fig. 5). In the Harfjärden population, mortality of eggs and embryos was higher in brackish water than in the freshwater treatment in all families, although some families produced viable larvae also in brackish conditions. In the Lervik population there was a more pronounced variation in how hatching success responded to salinity among families across the entire salinity gradient, compared with Harfjärden (Fig. 5). Genotype by environment interaction effects on body length of fry at the end of the yolk sac phase were evident within both populations (Table 1).

**Table 1:** Results from analysis of within population diversity in norms of reaction in response to salinity in two subpopulations of *Esox lucius*. Table shows family-by-salinity interaction (GxE) effects on hatching success of eggs and on fry body length in the population from Harfjärden and Lervik. Covariance parameter estimates were obtained from general linear mixed model analysis of variance. Statistical significance was assessed using the Wald Z-test.

Population	Trait	Covariance parameter estimate ± s.e.	*Z*	*P*
Harfjärden	Hatching success	0.0225 ± 0.00958	2.35	0.0095
	Fry body length	0.834 ± 0.3410	2.45	0.0072
Lervik	Hatching success	0.0591 ± 0.02236	2.64	0.0041
	Fry body length	0.318 ± 0.1127	2.82	0.0024

Results from analysis of covariance confirmed that total variance in hatching success among and within families was significantly greater in the Lervik population than in the Harfjärden population (ANCOVA, effect of population: *F*_1,7_ = 9.38, *P* = 0.018; effect of salinity: *F*_1,7_ = 0.0, *P* = 0.962) (Fig. 5). Total variance among and within families in body length of fry at the end of the yolk sac phase decreased with increasing salinity in Harfjärden (*F*_1,2_ = 34.99, *P* = 0.027) but not in Lervik (*F*_1,3_ = 0.87, *P* = 0.42; effect of interaction between population and salinity: (*F*_1,4_ = 8.43, *P* = 0.044).

## Discussion

Environmental heterogeneity is an important driver of genetic and phenotypic diversity within and between populations. Overall, spatially heterogeneous but temporally stable environments should result in divergent evolution of local adaptations and a high degree of specialism. Temporally unpredictable environments may instead contribute to the maintenance of a diversity of specialists, promote the evolution of generalist strategies, or favour diversified bet hedging strategies^13,14^. We evaluated these predictions by comparing salinity tolerance between two anadromous subpopulations of northern pike, *Esox lucius*, in the Baltic Sea that utilize freshwater spawning sites that differ regarding temporal variation in salinity.

The main findings and conclusions can be summarized as follows. (*i*) Effects of salinity were apparent on all investigated aspects of performance (albeit with the effect on days to hatch falling just short of statistical significance, *P* = 0.0515), and with the effects of salinity differing both among families and between populations, depending on performance measure. (*ii*) The two populations performed equally well in freshwater but responded differently in hatching success to higher salinity treatments, showing signs of divergent evolution. (*iii*) The Harfjärden population showed signs of specialization for freshwater spawning in the hatching phase, suggesting it had retained the ancestral state for this freshwater species. (*iv*) In the Lervik population early embryonic development showed higher tolerance to higher salinities, producing more than twice as many embryos in all saline treatments compared with the Harfjärden population. This broad tolerance is likely a result of occasional exposure to brackish water in the Lervik wetland. (*v*) There were differences between populations in variability (among families) of reaction norms linking hatching success and fry body length to salinity (GxE). Total variance among and within families was higher in Lervik.

Collectively, results suggest that spatial and temporal variation in salinity between and within the spawning sites has resulted in adaptive divergence of salinity tolerance during early embryonic development between populations. Additionally, the effects of salinity on total variance in hatching success and body length of yolk sac larvae differed both among and within families in these anadromous subpopulations of pike.

### Comparisons with other systems, species and populations

Salinity influences physiological processes of aquatic organisms and is a key environmental factor in structuring species distributions of fish^18^. Intraspecific variation in salinity tolerance has received considerable interest^46–48^. For example, comparisons among and within populations along the relatively stable saltwater gradient of the Baltic Sea have contributed important insights^49,50^, and point to salinity as a driver of local adaptation^47,51^. However, few studies have investigated the role of episodic selection associated with short-term changes in salinity (spikes), such as those caused by brackish water intrusions in stream mouths mediated by water level fluctuations, in shaping adaptations and within population variation (see^46,52^ for related examples in other systems). As such, the rare findings of the present study represent an important contribution towards our understanding of the evolution of salinity tolerance during early life stages. This is increasingly important in times when climate projections suggest pronounced alterations of salinity regimes in aquatic environments, not the least in the Baltic Sea^18,46,53^.

The salinity tolerance seen in the anadromous pike populations investigated in the present study differ somewhat from the findings in previous studies of populations that spawn in brackish water^25,33^. For example, Jørgensen, *et al*.^33^ show that a brackish spawning pike population that resides permanently in the Baltic Sea is unable to produce offspring in freshwater but can produce viable fry in salinities up to at least 8.5 psu. As we now have seen, fertilization, embryonic development, and successful hatching can occur in salinities up to at least 9 psu, even in *E*. *lucius* populations that spawn in freshwater wetlands (Figs 3 and 4). Discrepancies between studies and populations can probably be attributed in part to differences in environmental heterogeneity and specialization.

Does coping with a changing salinity regime come at a cost of maladaptation to freshwater environments? We observed tolerance to higher salinities in the Lervik than in the Harfjärden population. However, there was no sign of impaired performance at 0 psu, suggesting that there was no loss of adaptation to freshwater spawning in Lervik. Backflow of brackish water into the Lervik wetland is occasional, resulting in temporal variation in the direction and intensity of selection^6^. In combination with the potentially long life span, iteroparous reproductive strategy, and overlapping generations of pike^27,28^ this temporal heterogeneity and fluctuating selection regime should provide broad conditions for genetic polymorphisms^6,54,55^. Although salinity fluctuates also at Stege Nor it was seldom less than 7 ppt^34^. Thus, brackish coastal areas are relatively stable, and this may have favoured specialization towards spawning in brackish water for the population studied by Jørgensen, *et al*.^33^, particularly if there is no immigration and gene flow from populations spawning in freshwater environments. The loss of ability to produce viable offspring in freshwater may also reflect a loss of ancestral plasticity after enough generations of isolation. That the Lervik population maintained high performance in freshwater could be a reflection of a potentially more recent evolutionary split from the freshwater ancestor.

### On the effects of salinity on larval growth

In the Lervik population, body length of fry at the end of the yolk sac phase decreased with increasing salinity. A possible explanation for this finding is that higher levels of salinity in the external medium required energetically costly physiological adjustments^48,56,57^. It might be argued that brackish water incurs physiological costs regardless of reproduction mode, because it has been shown to stunt fry growth even in brackish spawning pike populations^33^. However, no consistent salinity-induced decrease in body length was evident in the Harfjärden population. The result was not a consequence of lower statistical power owing to extensive mortality and lack of data on body length at the highest salinity (9 psu) (Fig. 4). Indeed, in the Harfjärden population surviving fry were largest at 7 psu and there was a statistically significant effect of the interaction between population and salinity on fry size. We are unable to identify the mechanism(s) responsible for the relatively large body size at 7 psu in Harfjärden based on the data at hand.

When the results for Lervik and Harfjärden are compared it seems that coping with salinity during early embryonic and larval stages comes at the cost of impaired growth. Lervik embryos tolerated higher salinities better than Harfjärden embryos, but at the highest salinity treatment in which individuals from both populations produced viable progeny (7 psu) fry from Lervik were considerably smaller at the end of the yolk sac phase than fry from Harfjärden (ca 11 mm versus 13.5 mm) (cf Figs 3a and 4). The smaller size of the fry from Lervik may also reflect a cost of maintaining ancestral placidity that allowed them to tolerate and colonize different salinities.

### Causes of variation in salinity tolerance

Our results suggest that salinity tolerance during early development in anadromous pike populations have been shaped by a combination of spatial and temporal environmental heterogeneity. The wetland used for spawning by the Harfjärden population constitutes a relatively constant freshwater environment in that the altitudinal difference makes exposure to brackish waters very improbable given current sea levels (Fig. 1). This temporally stable environment should favour specialization towards freshwater spawning and potentially reduce genetic diversity in salinity tolerance. In agreement with this prediction, most genotypes in the Harfjärden population performed best in freshwater and showed diminishing success at higher salinity (Figs 3 and 5). Conversely, the Lervik wetland constitutes a temporally less stable spawning environment due to occasional brackish water backflow, which should favour generalization. In agreement with this expectation, we instead see a broad tolerance at the population level (Fig. 3a and b), with different genotypes having performance peaks spanning across almost the entire salinity gradient (Fig. 5b). Furthermore, total variance in hatching success was larger in Lervik compared with Harfjärden. This supports the interpretation that the greater population level generalism in Lervik reflects a mixture of individuals (genotypes) that employ different specialist strategies, rather than a common “jack-of-all-trades and master-of-none”-strategy employed by most genotypes^13,14^.

### Consequences of variation in salinity tolerance

High functional genetic diversity and the capacity for phenotypic plasticity is key to coping with challenges imposed by environmental change, including global warming^9,17,58^. The coastal fish community in the Baltic Sea is challenged by overfishing, habitat modifications, and environmental changes associated with climate change^28,59^. Hydrographic scenarios for the Baltic Sea coastal area forecast a few degrees higher temperature, increased precipitation, and a rise in sea levels over the next hundred years. This will drive the freshwater gradient to the south and thereby expand low salinity coastal habitats^53,60^. As a consequence of sea levels rising, there will be an increase in the number of wetlands used as spawning areas by pike influenced by backflow of brackish water. It is difficult to foresee how these long-term temperature and salinity changes will influence coastal fish populations and ecosystems^16,20,28^, and further research is needed.

## Conclusions

Some subpopulations of pike harbour large genetic variation in salinity tolerance and appear to be preadapted to future changes in salinity regimes. High functional genetic diversity increases establishment success in novel areas and the aptitude by which recently founded populations can adapt to new conditions by means of evolutionary modifications, ultimately resulting in range expansions^61–63^. *E*. *lucius* is invasive in some parts of North America including Alaska^64^ and it occurs in Europe from northern Italy in the south to Murmansk in northern Russia, spanning ca 24° in latitude^26–28^.

Our findings further emphasize that genetic structure and distinct subpopulations can occur at very small spatial scales, even in organisms that live sympatrically and share a common habitat for foraging and growth during most of their life. The results of the present and earlier studies of anadromous pike^28,37–39^ underscore that it is essential in population genetics studies to take into consideration the behaviour and life-history of the species when designing on a sampling scheme, else results and conclusions may be misleading.

Our present results provide insights of relevance from applied perspectives regarding the conclusions that can or cannot be made based on estimates of functional as opposed to neutral genetic diversity. Our split brood experiment demonstrated that variation in hatching success among families was greater in Lervik than in Harfjärden, indicative of higher genetic variance in salinity tolerance. By contrast, neutral genetic diversity as estimated based on microsatellite data was lower in Lervik than in Harfjärden (see Methods). Together, these findings add to the body of evidence that estimates of neutral genetic diversity (based on e.g., microsatellites) cannot be used as substitutes for functional (e.g., salinity tolerance) diversity to infer evolutionary potential and the ability of populations to cope with environmental change^65–67^. It is imperative that future studies also further investigate functionally informative population proxies, so that conservation efforts can be decided based upon functional genetic diversity.

### Ethical approval

The study was carried out in accordance with all relevant applicable national guidelines for the care and use of animals. Ethical approval for the study was granted by the Ethical Committee on Animal Research in Linköping, Sweden (approval Dnr ID 83). Permission for field studies was granted by the County Administrative Board in Kalmar (approval 623–1681–13).

## Electronic supplementary material

Supplementary Information
