# Author Correction: Variation in salinity tolerance between and within anadromous subpopulations of pike (*Esox lucius*)

**DOI:** 10.1038/s41598-018-24805-1

**Published:** 2018-04-26

**Authors:** Johanna Sunde, Carl Tamario, Petter Tibblin, Per Larsson, Anders Forsman

**Affiliations:** 1 Ecology and Evolution in Microbial Model Systems, EEMiS, Department of Biology and Environmental Science, Linnaeus University, SE-391 82 Kalmar, Sweden; 2 Present Address: Swedish University of Agricultural Sciences, Department of Aquatic Resources, SE-702 15 Örebro, Sweden

Correction to: *Scientific Reports* 10.1038/s41598-017-18413-8, published online 08 January 2018

The original version of this Article contained an error in the title of the paper, where the word “*lucius*” was incorrectly given as “*1ucius*”. This has now been corrected in the PDF and HTML versions of the Article.

