# Supplementary material for: Variation in salinity tolerance between and within anadromous subpopulations of pike (Esox lucius)
Source: Sci Rep. 2018 Jan 8;8:22. doi: 10.1038/s41598-017-18413-8 (PMC5758576; doi:10.1038/s41598-017-18413-8)
Supplement: Supplementary file 1 — Supplementary Information [file 41598_2017_18413_MOESM1_ESM.pdf]

Variation in salinity tolerance between and within anadromous subpopulations of pike (*Esox lucius*)

Johanna Sunde, Carl Tamario, Petter Tibblin, Per Larsson, and Anders Forsman*

* Author for correspondence E-mail: anders.forsman@lnu.se

Supplementary Information

SUPPORTING METHODS AND RESULTS

Evaluating assessment of fertilization success in brackish water. Eggs that fail to be fertilized during the activation window (according to Raat [1] micropyle stays open for approximately 1 minute) die, and dead eggs become milky and are easily discernible (**Figure S1a**). One of the aims at the onset of this study was to investigate whether and how salinity influences fertilization success. To evaluate whether it is possible to readily and accurately discriminate fertilized (and supposedly live) from unfertilized (and supposedly dead) eggs in a salinity gradient, a complementary incubation experiment was carried out.

Gametes from three female and one male pike were acquired from the Lervik population on the 15th of April, 2016, and brought to the laboratory in Kalmar. Eggs from each female were split into three fertilization treatments: (a) completely unfertilized and unstirred, (b) unfertilized but stirred as in the fertilization process, and (c) fertilized and stirred in accordance with the complete procedure. All females and all salinities (0, 3, 5, 7 and 9 psu) were represented in each treatment. The eggs from all females in treatment (c) were fertilized with milt from the same male. This was to avoid confounding effects of any variation in quality or viability among males and differences in fertilization success owing to male-female genetic incompatibility. Treatment (c) with fertilized eggs was included to establish whether the eggs were healthy and capable of producing live embryos, which they were.

The eggs were photographed (using Panasonic DMC-TZ5) one hour after initialization and then every day for 9 days. Results based on analyses of pooled data for unfertilized stirred (treatment a) and unfertilized unstirred (treatment b) showed that dead eggs (due to any unknown reason, e.g. failed fertilization or rough handling) do eventually turn white and milky even in brackish water, although the process is much slower compared to fresh water (**Figure S1b**). Because the timing of the morphological appearance of dead eggs differed between salinities, accurate assessments and comparisons of fertilization success through

inspection of eggs at an early stage was not possible. Counting milky eggs can potentially be used to assess fertilization success in experiments in which only freshwater is used. Egg swelling, another indicator of successful fertilization [1], could not be detected retrospectively by ocular inspection of eggs in photographs. For the reasons above, we were unable to investigate whether salinity influenced fertilization success in these populations. However, fertilization success has previously been reported not to be affected to any important degree by salinity in pike [1,2].

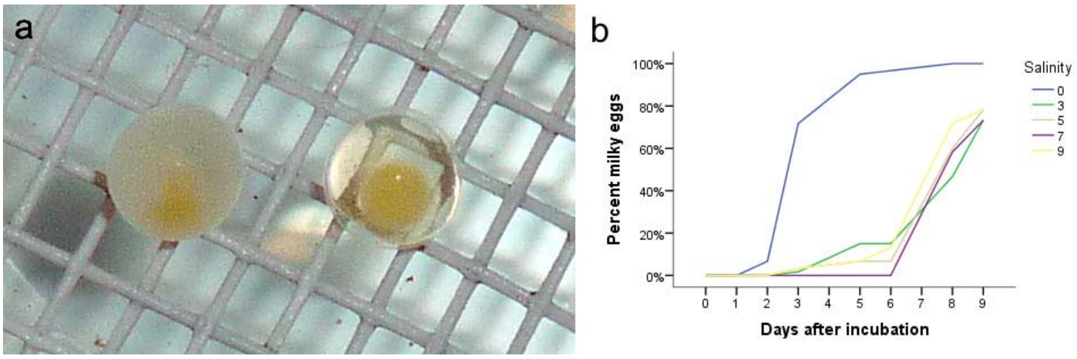

Supplementary Figure S1. Appearance of fertilized and unfertilized pike eggs. (**a**) The photograph shows the difference in appearance between a dead (left) and a live (right) pike *Esox lucius* egg. The photograph was taken one day after incubation. Photo by Carl Tamario. (**b**) The graph illustrates the rate at which unfertilized dead eggs change their appearance and become milky depending on salinity treatment.

Supplementary References

1 Raat, A. J. P. *Synopsis of Biological Data on the Northern Pike Esox lucius*. Vol. 30 Rev. 2 178 (Food and Agriculture Organisation of the United Nations Fisheries Synposis No. 30 Rev. 2., 1988).

2 Jørgensen, A. T. *et al.* High salinity tolerance in eggs and fry of a brackish *Esox lucius* population. *Fisher Managm Ecol* **17**, 554-560, doi:10.1111/j.1365-2400.2010.00755.x (2010).
